# Proteins differentially expressed in elicited cell suspension culture of *Podophyllum hexandrum* with enhanced podophyllotoxin content

**DOI:** 10.1186/1477-5956-10-34

**Published:** 2012-05-23

**Authors:** Dipto Bhattacharyya, Ragini Sinha, Srijani Ghanta, Amrita Chakraborty, Saptarshi Hazra, Sharmila Chattopadhyay

**Affiliations:** 1 Plant Biology Laboratory, CSIR-Indian Institute of Chemical Biology, 4, Raja S. C. Mullick Road, Kolkata, 700032, WB, India

**Keywords:** MeJA elicitation, Podophyllotoxin, *Podophyllum hexandrum* Royle, Differential proteomics, MALDI TOF-TOF MS/MS

## Abstract

**Background:**

Podophyllotoxin (PTOX), the precursor for semi-synthesis of cancer therapeutics like etoposide, teniposide and etophos, is primarily obtained from an endangered medicinal herb, *Podophyllum hexandrum* Royle. PTOX, a lignan is biosynthetically derived from the phenylpropanoid pathway. The aim of this study is to investigate changes in the *P. hexandrum* cell proteome potentially related to PTOX accumulation in response to methyl jasmonate (MeJA) elicitation. High-resolution two-dimensional gel electrophoresis (2-DE) followed by colloidal Coomassie staining and mass spectrometric analysis was used to detect statistically significant changes in cell’s proteome.

**Result:**

The HPLC analysis showed approximately 7–8 fold change in accumulation of PTOX, in the 12day old cell suspension culture (i.e. after 9days of elicitation) elicited with 100 μM MeJA as compared to the control. Using 2-DE a total of 233 spots was detected, out of which 105 spots were identified by MALDI TOF-TOF MS/MS. Data were subjected to functional annotation from a biological point of view through KEGG. The phenylpropanoid and monolignol pathway enzymes were identified, amongst these, chalcone synthase, polyphenol oxidase, caffeoyl CoA 3-O-methyltransferase, S-adenosyl-L-methionine-dependent methyltransferases, caffeic acid-O-methyl transferase etc. are noted as important. The relation of other differentially accumulated proteins with varied effects caused by elicitors on *P. hexandrum* cells namely stress and defense related protein, transcription and DNA replication and signaling are also discussed.

**Conclusions:**

Elicitor-induced PTOX accumulation in *P. hexandrum* cell cultures provides a responsive model system to profile modulations in proteins related to phenylpropanoid/monolignol biosynthesis and other defense responses. Present findings form a baseline for future investigation on a non-sequenced medicinal herb *P. hexandrum* at molecular level.

## Background

**Figure 1:**
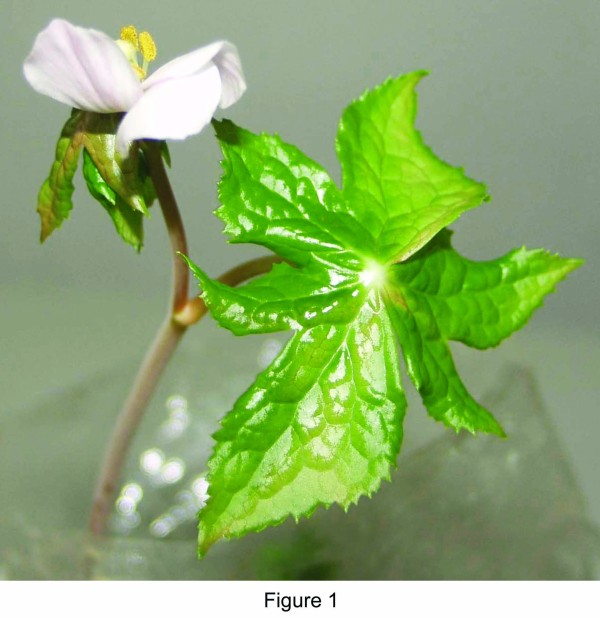
**A flowering twig of *Podophyllum hexandrum. ***

*Podophyllum hexandrum* Royle, commonly referred to as the Himalayan Mayapple, is an endangered perennial herb belonging to the family Berberidaceae that is distributed on the lower slopes of the Himalayas in scrub and forest, from Afghanistan to central China [1]. Roots and rhizomes of *P. hexandrum* contain lignans such as PTOX and other related aryltetralin lignans [2]. Till date, PTOX has been used as the starting compound for the production of the semi-synthetic drugs etoposide (VP-16-213), teniposide (VM-26) and ethophos, which are used in the treatment of lung and testicular cancers [3], leukaemia and rheumatoid arthritis [4]. The Indian species *P. hexandrum* (Figure 1) contains three times more PTOX than its American counterpart *P. peltatum*, which contains other lignans viz. α- and β-peltatins [5,6]. However, peltatins do not contribute to the anti-cancer properties of the plant [7]. To meet the commercial demand, up till now PTOX has been extracted from the rhizomes of *P. hexandrum* and *P. peltatum* collected in the wild; chemical synthesis of PTOX is possible but not economically feasible [8]. Therefore, rhizomes are indiscriminately collected in large quantities to meet the ever-increasing demand for the drug in modern medicine. Severe habitat destruction and over-collection has created an acute depletion in the population of this herb. Together with a lack of organized cultivation, this has led to *P. hexandrum* being classified as a critically endangered species of the Himalayan region [9,10].

**Figure 2:**
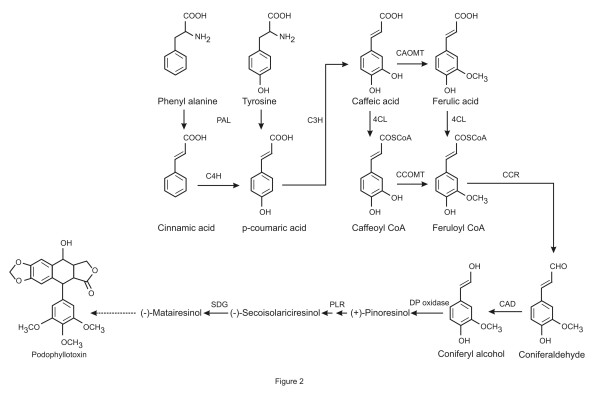
**Schematic representation of podophyllotoxin biosynthetic pathway.** Dashed arrows represent unknown multiple reactions. Enzyme abbreviations: PAL– Phenylalanine ammonia lyase, C4H– Cinnamate 4-hydroxylyase, C3H– p-coumarate 3-hydroxylyase, CAOMT– caffeic acid–O-methyltransferase, 4CL– 4coumarate: CoA ligase, CCOMT– caffeoyl CoA o-methyltransferase, CCR– cinnamoyl CoA reductase, CAD- cinnamyl alcohol dehydrogenase, DP oxidase– dirigent protein oxidase, PLR– pinoresinol lariciresinol reductase, SDG– Secoisolariciresinol dehydrogenase.

PTOX is derived biosynthetically from the phenylpropanoid pathway by the dimerization of two phenylpropanoid units [11]. The biosynthesis of the phenylpropanoid unit starts with the aromatic amino acid phenylalanine, which is deaminated by the phenylalanine ammonia-lyase (PAL) to cinnamic acid. Cinnamic acid is channeled through the general phenylpropanoid pathway before entering the monolignol specific branch. This branch includes two enzymatic reactions—a reduction step catalysed by the cinnamyl-CoA:NADP oxidoreductase and a dehydrogenation step catalyzed by cinnamyl alcohol dehydrogenase (CAD)— producing the cinnamyl alcohols, also called monolignols. Dirigent mediated coupling of two molecules of coniferyl alcohol produces pinoresinol, which is the precursor of the lignans PTOX and other lignans as well (Figure 2).

*In vitro* production of PTOX through cell culture of *Podophyllum* spp. has been reported previously [12-16]. In addition to *Podophyllum* spp., a number of other plants including *Linum album, Juniperous chinensis* and *Callitris drummondii* have been investigated for the *in vitro* production of PTOX and its derivatives [17-19]. However, the production of PTOX using cell cultures may not be sufficient for biotechnological production systems [20]. Elicitation is an approach that may overcome the limitations of the *in vitro* culture system.

In general, elicitation experiments have two main goals. The first is the enhancement of secondary metabolite production for commercial use. The second goal is to gain more insight into the biosynthetic pathways leading to the formation and regulation of secondary metabolites. There are many reports showing enhancement in the level of PTOX following MeJA elicitation [21,22]. MeJA has also been used to obtain enhanced production of PTOX in embryogenic cell suspension cultures of *P. peltatum*[23]. Using elicitor-induced plant cell cultures, Suzuki and co-workers [24] showed that a large number of genes involved in secondary metabolism including L-phenylalanine ammonia-lyase, cinnamate 4-hydroxylase, caffeic acid 3-*O*-methyltransferase and chalcone synthase were differentially regulated in response to different elicitors in *M. truncatula.* Elicitors activate plant natural defense responses, including increased secondary metabolite production. In this investigation, this elicitation strategy has been exploited to obtain enhanced PTOX accumulation in the cell suspension culture of *P. hexandrum*, with a view to explore the cell’s proteome having enhanced level of PTOX.

Proteomic studies in plant systems have primarily been performed in fully sequenced model systems such as *Arabidopsis thaliana*, *Oryza sativa* (rice), *Populus trichocarpa* (black cottonwood), and *Vitis vinifera* (grape vine) since mass spectrometry (MS)-based proteomics requires the availability of a protein database [25]. Relatively few studies have applied proteomics for investigating secondary metabolism of medicinal plants [26-28], and in particular have focused on applying proteomics for discovering new enzymes involved in secondary metabolism [29,30]. The present work was undertaken to explore protein profile of elicited cell suspension culture of *P. hexandrum* resulting in enhanced accumulation of PTOX. To accomplish this aim, 2-DE proteomic profiling of *P. hexandrum* cell suspension cultures elicited with MeJA resulting in enhanced PTOX content along with control culture devoid of MeJA was performed which provided clear information regarding the differential protein abundance. MALDI TOF-TOF MS/MS analysis was performed for protein identification. Data were subjected to functional annotation from a biological point of view through KEGG. This investigation is an attempt on proteomics analysis of *P. hexandrum*, an endangered non-sequenced medicinal herb towards future exploitation.

## Results

### MeJA elicitation and PTOX accumulation

**Figure 3:**
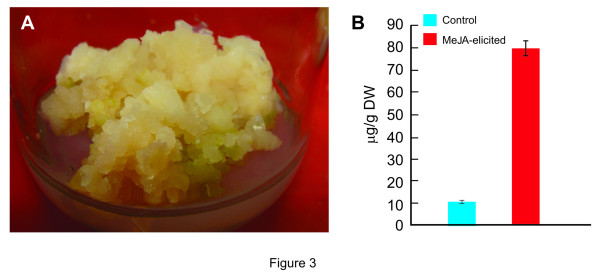
(**a**) **Callus of *P. hexandrum *used for the initiation of cell suspension culture.** (**b**) Estimation of podophyllotoxin in control and MeJA elicited cell suspension cultures of *P. hexandrum* using HPLC analysis. Data are the mean ± SD for three individual experiments (n = 3).

The cell suspension culture of *P. hexandrum* leaf-derived calli (Figure 3a) was elicited with various concentrations of MeJA (10–100 μM). The HPLC analysis showed approximately 7–8 fold change in accumulation of PTOX, in the 12day old cell suspension culture (i.e. after 9day of elicitation) elicited with 100 μM MeJA as compared to the control (Figure 3b) which can be corroborated with earlier report [22]. The fractions collected from HPLC were subjected to mass spectrometry (LC-MS/MS) for the identification of PTOX (see Additional file 1). A time dependent analysis (6 to 72 h after elicitation) of PTOX accumulation at same concentration of MeJA was also performed, however insufficient accumulation of PTOX was observed in elicited culture as compared to the control (data not shown).

### Protein abundance pattern of *P. hexandrum* cell culture upon elicitation

**Figure 4:**
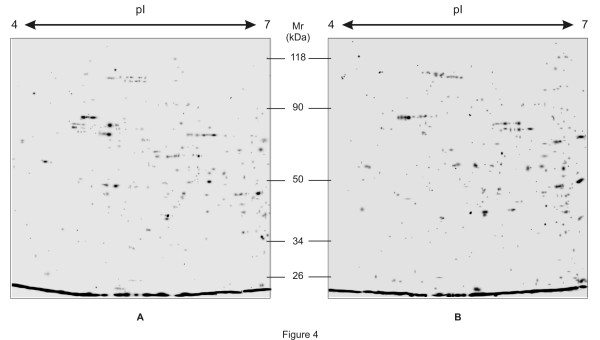
**Representative two-dimensional gel electrophoresis of total soluble proteins extracted from (a) control and (b) MeJA-elicited cell suspension cultures of *P. hexandrum.***

Elicitation of *P. hexandrum* cell suspension culture with MeJA led to the modification of its proteome profile. 2-DE was performed in triplicate to study the abundance patterns of proteins extracted from control and elicited cultures (9day after elicitation with 100 μM MeJA). The experiment was repeated with three batches of independent cultures for control and elicited samples. Owing to sample limitation and economical issues, technical replicates could not be prepared. The three biological replicates of protein gels for each treatment were used for further analysis. Figure 4a and 4b shows the representative 2-DE gel images of control and elicited samples, respectively. Immobilized pH gradient (IPG) strips (18 cm, pH 4–7) and colloidal Coomassie stain was used. The protein yield of the employed protocol for protein isolation was very similar in both control (3.56 ± 0.36 μg of protein/mg of fresh tissue) and elicited (3.28 ± 0.62 μg of protein/mg of fresh tissue) cultures. Table 1 shows the number of total spots detected (415 ± 5 in control and 492 ± 8 in elicited culture, respectively) and the number of variable (those that were qualitatively and quantitatively different as compared to control) spots found after 2-DE of protein extracts from control and elicited culture. 337 spots were common to both the samples, while out of 233 differentially abundant spots, 78 and 155 spots were detected in control and elicited cultures, respectively, the mean coefficient of variation being 34.6%.

**Table 1:** **Summary of the detected spots in control and elicited cultures of *P. hexandrum *used for proteome analysis**

**Sample**	**Protein yield (μg/mg of fresh tissue)**	**No. of spots (mean ± SD)**	**No. of variable spots w.r.t**^***a***^** control**	**Qualitative difference w.r.t control**	**Quantitative difference w.r.t**^**a**^** control**		
				**Newly appeared**	**Disappeared**	**Up accumulated**	**Down accumulated**
**Control**	3.56 ± 0.36	415 ± 5	-	-	-	-	-
**Elicited**	3.28 ± 0.62	492 ± 8	155 ± 2	82 ± 3	47 ± 1	16 ± 4	10 ± 1

^*a*^ with respect to.

A set of differentially abundant protein spots was selected from control and elicited cultures. For that purpose, differences in spot abundance was statistically evaluated using the *t*-test function implemented in the PD Quest software. Means and standard deviations were calculated from three independent sets of treatments (biological replicates). Spots showing differences between control and elicited cultures with a P < 0.05 at any of the time points were chosen for further analysis.

### Identification of differentially expressed proteins

**Figure 5:**
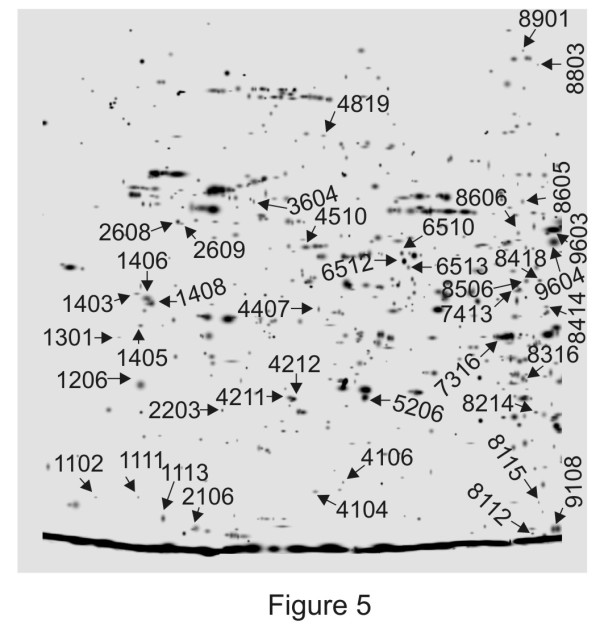
**Annotated gel image representing predicted functions of proteins.** Master gel combining spots of control and MeJA-elicited cell culture protein extracts. Some of the representative spots of major categories are annotated corresponding to their respective SSPs as listed in Additional file 2.

**Figure 6:**
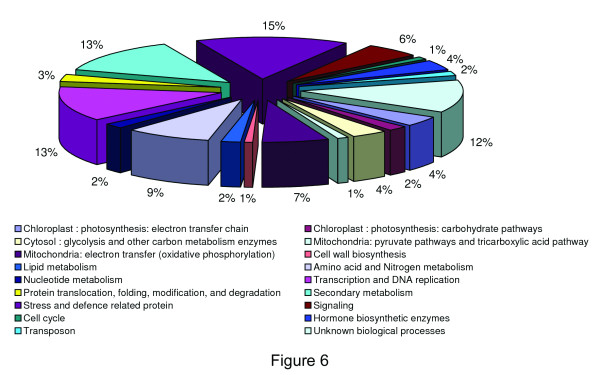
**Putative functional classifications of differentially accumulated proteins in MeJA elicited cell suspension cultures of *P. hexandrum ***.

Out of the 233 differentially abundant spots, 105 spots were significantly identified through MALDI TOF-TOF MS/MS. Only the spots having MASCOT score ≥ 60 (P < 0.05) were considered as confident identification. Additional file 2 summarizes only the 105 identified proteins differentially accumulated in the elicited culture. Additional file 3 shows the sequences of all the identified peptides with the corresponding ion score in brackets that were matched based on the MS/MS patterns. Some of the representative spots of major categories are annotated in Figure 5, the arrows and numbers refer to the spots exhibiting significant changes in relative spot abundance after elicitation, corresponding to their respective SSPs (see Additional file 2). The 105 identified protein sequences were classified into 18 categories according to KEGG [31], based on putative functions of secondary metabolism (14 proteins), stress and defense related protein (16 proteins), transcription and DNA replication (14 proteins), signaling (6 proteins), the principal groups found. The functional distribution of the proteins is represented in Figure 6.

Some of these proteins were present in more than one spot. This co-migration of proteins is possible if both present the same experimental pI and Mr. The presence of the same protein in more than one spot [i.e. photosystem I reaction centre subunit PSAN precursor, aspartate aminotransferase, F-box family protein, pentacotripeptide repeat (PPR) containing protein, chalcones synthase, serine/threonine specific protein kinase, NBS/LRR resistance protein like protein, pathogenesis related protein-10-3.1, phytocalpain] has been reported. While for most of the identified proteins close theoretical and experimental Mr values were obtained. For others, lower experimental Mr than theoretical values were obtained, these probably being degradation products of the native protein (i.e. isocitrate lyase, caffeic acid O-methyl transferase, putative benzothiadiazole-induced S-adenosyl L-methionine salicylic acid carboxyl methyl transferase, NBS/LRR resistance protein like protein, pathogenesis related protein-10-3.1, phytocalpain, inositol polyphosphate 5-phosphatase). In other cases there were only small differences in pI described, suggesting the existence of post-translational modifications or isoforms. There were another sets of proteins in which the pI and Mr value were not coincident with the heterologous sequence in the database. Wan and Liu [32] reported that the apparent Mr values predicted by SDS-PAGE had an error deviation of about ± 10% compared with the theoretical values. In most of the cases, this was because only a fragment of the protein sequence was included in the database. Isoforms of some protein like pathogenesis related protein were identified in more than one spot. This may be explained by different splicing variants, or posttranslational modifications or cleaved isoforms of the same protein [33].

## Discussion

A search was made for proteins differentially accumulated in cell suspension cultures of *P. hexandrum* elicited with MeJA resulting enhanced accumulation of PTOX. The ability to perform proteomic analyses of medicinal plants without a fully sequenced and annotated genome will provide a useful platform and form a baseline for exploring and investigating the complex physiological pathways related to metabolism, defence and signaling of these medicinal plants. In this investigation 100 μM MeJA was noted as optimum for enhanced accumulation of PTOX which can be corroborated with previous report where 2 to 10- fold enhancement of PTOX was noted in cell suspension culture of *L. album*[22]. The role of MeJA in triggering phenyl propanoid biosynthetic pathway and elicitation of defense related proteins are the major effects described previously. Thus, our analysis is focused on proteins closely related to these effects. However, proteins related to other categories have also been discussed in brief.

### Proteins related to secondary metabolism

The major category of the identified protein was related to secondary metabolism comprising of 13% of the total proteins identified. This is in consistence with previous reports of elicited cell cultures in which several secondary metabolic enzymes were identified [28]. However, the proteomes of a few medicinal plants with rich but incomplete sequence information have been explored, including *Taxus cuspidate*[26], *Catharanthus roseus*[27], *Papaver somniferum*[28,34], ginseng [35] and *Chelidonium majus*[36]. For proteomic analysis of such organisms, one approach utilizes sequence homology to proteins already in a database. This approach allows identification of highly conserved proteins such as primary metabolic pathways proteins but not necessarily enzymes involved in specific secondary metabolic pathways like PTOX biosynthesis which are specific to individual plant species. Elicitor-mediated accumulation of phenylpropanoid compounds in plant cell cultures has been studied in greatest detail in parsley, chickpea, soybean and alfalfa cells [37-40]. In our study also several proteins related to phenylpropanoid biosynthetic pathways were identified, which is consistent with previous studies, for example- Chalcone synthase, it plays an essential role in the biosynthesis of plant phenylpropanoids and catalyzes the first step in flavonoid biosynthesis [41]. Likewise, plant *O*-methyltransferases (OMTs) constitutes a large family of enzymes that methylate the oxygen atom of a variety of secondary metabolites including phenylpropanoids, flavonoids, and alkaloids. *O*-Methylation plays a key role in lignin biosynthesis, stress tolerance, and disease resistance in plants [42]. Monolignol pathway enzymes like Caffeoyl CoA 3-O-methyltransferase (CCOMT), a class A O-methyltransferase was found to be up-accumulated in elicited culture [43] and is essential for the biosynthesis of coniferyl and sinapyl alcohols, the precursors of lignins and lignans [44,45]. Likewise S-adenosyl-L-methionine-dependent methyltransferases (SAM-methyltransferases) and caffeic acid-O-methyl transferase (CAOMT) identified as up-accumulated in the present study are the key enzymes in phenylpropanoid, flavonoid and monolignol pathway [46,47]. The identification of these methyltransferases, suggests the probability of the presence of such homologous proteins in *P. hexandrum*, which may have a role in PTOX biosynthetic pathway.

The biosynthetic pathway of PTOX is yet to be revealed to its full extent, however the identification of several monolignol as well as phenylpropanoid pathway related enzymes, after MeJA-elicitation substantially adds to the available information. This approach may be used to identify and trace the poorly categorized pathway of PTOX. Similarly differential expression of proteins related to other secondary metabolic pathways were also observed indicating the enhancement of homologous proteins related to PTOX biosynthetic pathways following the MeJA elicitation. Proteins such as polyphenol oxidase was identified as well, these have diverse roles related to secondary metabolism as well as defense, induction of these proteins following wounding and elicitations have been reported vividly [48]. Proteins related to other secondary metabolic pathways like anthocyanin pathway such as chalcone-flavone isomerase and resveratrol synthase, involved in synthesis of resveratrol (phytoalexin) were also observed which may be due to MeJA elicitation as reported previously [49]. Likewise dioxygenases and tyrosine decarboxylase were also identified, these are important in the biosynthesis of plant signaling compounds such as abscisic acid, gibberellins, and ethylene and also of secondary metabolites, notably flavonoids and alkaloids [50,51].

### Stress and defense related protein

Jasmonates are known as defense gene regulators in virtually all plant systems. The MeJA elicited culture also showed an up-accumulation of defense/stress-related enzyme, the second major category of the identified protein, which forms 15% of the total proteins identified. Pathogenesis related proteins Pr-1 like protein and intracellular PR-104 proteins were up-accumulated in the elicited cells, which can be correlated with previous reports [52,53]. In general, pathogenesis related proteins are induced by pathogen challenge and exhibit antibacterial and antifungal activities [54]. Although PR-10 are classified as defense response proteins, however, the down-regulation of PR-10 was observed in present study, which suggests that they may be involved in functions different from defense, for example some have ribonuclease activity and ligand binding capacity [55] as well, but its precise function is still unclear. Similarly a downregulation of alcohol dehydrogenase was observed. Likewise the identification of other proteins like glutathione transferase, benzothiadiazole-induced S-adenosyl L-methionine salicylic acid carboxyl methyl transferase, NBS/LRR resistance protein and LRR kinase protein as a defense response following elicitation is also a well known fact and may be correlated with previous studies [49,56]. The ability of jasmonate to induce variety of defence related proteins reported previously can be corroborated with our investigation [57-59].

### Signaling

Most proteins identified as signal transduction components belong to calcium signaling pathways and include a calmodulin, which increased in abundance in response to elicitor treatment. Calcium flux is important for the elicitor-induced accumulation of secondary metabolites [60] and calmodulin is a ubiquitous calcium sensor. Calmodulin has been implicated in the salicylic acid-independent defense response in tobacco [61]. Phosphoinositide specific phospholipase C family protein was found to be down-regulated. Binding of TFs to DNA can be regulated via protein phosphorylation and dephosphorylation which in turn regulate expression of many target genes, including TF genes [62]. Therefore, regulation of TF biosynthesis, activation, and inactivation provides a flexible network for regulation of plant secondary metabolites.

### Transcription and DNA replication

It is believed that all signal transduction pathways finally converge on transcription factors (TFs), and almost all genes for secondary metabolite biosynthesis are regulated by specific TFs. Identification of TFs like Apetala 1 can be corroborated with previous reports where rapid accumulation of AP2/ERF-domain TFs in response to fungal elicitor or MeJA treatment has been shown [63]. Many genes in the phenylpropanoid pathway possess the same TF-binding cis-elements and are regulated by the same TFs. Similarly role of cis acting elements such as MYB TFs in binding to specific phenylpropanoid pathway genes has also been reported previously [64]. Myb and bHLH, essential for regulating phenylpropanoid and flavonoid biosynthesis pathway, can physically interact with each other [65]. Role of R2R3 Myb transcription factor in MeJA signal transduction and induction of phenylpropanoid biosynthetic pathway genes is also reported [66] indicating the essential roles of TFs in regulation of plant polyphenolics like lignans. Down-regulation of certain transcription factors like F-box proteins has been observed. Role of these proteins has been observed in various developmental processes in plants, for example photomorphogenesis, circadian clock regulation, self incompatibility, and floral meristem and floral organ identity determination, involves F-box proteins [67-69].

### Proteins related to other metabolic pathways

The *P. hexandrum* cell suspension cultures were induced from leaves therefore spots corresponding to proteins related to various categories like electron transfer chain, carbohydrate pathways, glycolysis, pyruvate pathways, tricarboxylic acid pathway and other carbon metabolism enzymes were also identified. Significant down-regulation of some of these proteins like galactonolactone dehydrogenase, quinone oxidoreductase and inositol polyphosphate 5 phosphatase were observed. Proteins related to other biological functions like protein translocation, folding, modification, and degradation were also identified. Previous work of cell proteome elicited with fungal elicitors has also shown similar proteins [27,28], which can be correlated with our study.

Proteins related to other categories like cell wall biosynthesis, lipid metabolism, nucleotide metabolism, amino acid metabolism was observed. Down regulation of some of these proteins like aspartate aminotransferase was noted. Interestingly cyclin A like protein involved in cell cycle was observed to be down regulated which may be correlated with previous study of significant decrease in cell growth following MeJA elicitation [70,71]. Hormone biosynthetic enzymes like 12-oxophytodienoic acid reductase, involved in the biosynthesis of jasmonic acid, a signaling molecule commonly induced in response to stress, wound, and pathogenic and herbivore attacks and was identified in our study and in a proteomics survey of a *C. roseus* cell culture producing monoterpenoid indole alkaloids. Jasmonic acid is a critical signaling molecule in plant defense responses [60]. Rapid accumulation of endogenous jasmonic acid in response to elicitation has been noted in many plant cell cultures of diverse taxonomic origins, and it has been suggested to mediate the induction of many defense-responsive natural products. In general, identification of proteins related to diverse metabolic pathways following MeJA elicitation can be corroborated with previous reports [49]. However, less is known about how primary and secondary metabolic pathways are coordinated to support increased secondary metabolite production. Proteins related to transposon and unknown biological processes were also observed (see Additional file 2). The sequence information on *P. hexandrum* is unavailable and the number of functionally annotated ESTs is low at this time. As this number increases, the proteome data will be proven even more useful. In the existing databases, the proteins of unknown function are denoted as unknown protein, hypothetical protein or putative protein. The hypothetical proteins are peptides generated by computer assisted theoretical translation of nucleotide sequences but have never been identified as protein products.

Understanding the temporal, spatial, developmental, and environmental expression of proteins is useful for further analysis of its function in any associated condition. The use of protein profiling, identification of protein classes and their expression profile at subcellular level define the functions involved in metabolic pathways, which ultimately dictate the cellular physiology. Thus, assigning a functional role to each of the yet unidentified protein will be challenging and quite valuable in order to know their physiological significance in *P. hexandrum*.

## Conclusions

Proteomics research not only provides new insights into protein expression patterns, but also enables identification of many attractive candidates for further investigation. Elicitor-induced PTOX accumulation in *P. hexandrum* cell culture provides a responsive model system to profile modulations of altered proteins. Here, 2-DE combined to mass spectrometry led to the identification of several different functional categories of proteins like-secondary metabolism, stress and defense, transcription and DNA replication and signaling. Thirteen percent of the identified proteins were related to secondary metabolism, identification of several enzymes related to monolignol/phenylpropanoid biosynthesis especially various methyl transferases like- CCOMT and CAOMT was observed. Furthermore, identification of these up-stream monolignol/phenylpropanoid pathway proteins also signifies their role in controlling the PTOX biosynthetic pathway. Hence, information generated in the present study may suggest that the biosynthesis of the PTOX could be regulated by the expression of upstream genes of monolignol pathway viz. CCOMT and CAOMT. Identification of several up-stream phenylpropanoid pathway proteins also supports this notion. However, more in depth investigation on high throughput analysis of proteins, and measurement of secondary metabolic intermediates will be essential for clear understanding of the pathway especially in a non-model endangered species like *P. hexandrum*. Dissection of the signaling network will also lead to discoveries of more secondary metabolite biosynthetic genes and regulatory factors, and will facilitate a specific and efficient engineering of the production of target secondary metabolites. In summary, results shown in this study provide valuable basic information of the cell proteome of *P. hexandrum* that resulted due to enhanced accumulation of PTOX, and offers interesting possibilities for future research.

## Methods

### Plant material and growth conditions

*Podophyllum hexandrum* Royle plants were procured from the Institute of Himalayan Bioresource Technology (a unit of CSIR), Palampur, Himachal Pradesh, India as a gift. Calli were induced from mature leaves in MS medium [72] supplemented with 2.68 μM NAA and 8.88 μM BAP [73]. Callus was subcultured after every two weeks in the above-mentioned medium. Cell suspension cultures were initiated from freshly subcultured brown friable calli of *P. hexandrum* in modified liquid MS medium [15] containing 60 mM total N_2_ content, 1.25 mM potassium dihydrogen phosphate, 6% glucose and 11.41 μM IAA. Finally, cultures were elicited with 10–100 μM MeJA (Duchefa, Germany) on the third day of initiation of culture. Each batch of cells was harvested nine days after elicitation. The cells were collected by centrifugation at 1000 × g for 10 min, rinsed five times with sterile distilled water and used for further study.

### Lignan extraction and HPLC analysis

Lignans were extracted from *P. hexandrum* cells [14]. In brief, 100 mg of control and treated cultures was extracted with 2 ml ethanol for 20 min at 60°C in microtubes and sonicated for 15 min. The supernatant was collected after centrifugation and evaporated to dryness under vacuum. Extracts dissolved in methanol were used for HPLC analysis. PTOX (Sigma, USA) was used as a standard. PTOX extractions were performed with three biological replicates.

HPLC was carried out with Waters 515 HPLC Pump and Waters 2998 photodiode-array detector. The system was controlled with Waters Pump Control Module II. Data analyses were performed with Empower 2 software. The detector was set at 290 nm and separation was carried out using an XTerra RP18, 5 μm, (4.6 × 250 mm i.d.) column. A guard column, XTerra RP18, 5 μm, was used to safeguard the analytical column. Chromatographic conditions were essentially as described earlier [6]. A linear gradient was applied with acetonitrile as solvent (A) and 0.01% (v/v) H_3_PO_4_ in water as solvent (B). From 0 to 17 min, (A) increased from 40% to 67% and the flow-rate increased from 0.8 ml min^-1^ to 1.0 ml min^-1^, from 17 to 18 min, (A) changed from 67% to 40% with 1.0 ml min^-1^ flow-rate, and from 18 to 24 min, (A) was 40% and the flow-rate decreased from 1.0 ml min^-1^ to 0.8 ml min^-1^. MS analysis was performed in ESI positive ion-mode with Micromass Q-T of microTM to determine the molecular weight of cell culture extracted PTOX and standard PTOX.

### Protein extraction

Proteomic studies were carried out as mentioned in [74]. In brief, 1 g of cells were ground to a fine powder under liquid nitrogen and suspended in extraction buffer (700 mM Sucrose, 500 mM Tris–HCl, pH 7.5, 50 mM EDTA, 100 mM KCl, 2% (w/v) β-mercaptoethanol, and 1 mM PMSF), and an equal volume of ice-cold Tris–HCl, pH 7.5 saturated phenol was added. The mixture was shaken at 4°C for 30 min. After centrifugation (5000 × g, 4°C, 30 min), the phenol phase was collected. This phenolic phase was extracted three times with extraction buffer. Protein was precipitated from the collected phenol phase by adding five volumes of 100 mM ammonium acetate in methanol and incubating overnight at −20°C. The protein pellets were rinsed twice with ice-cold methanol and acetone, respectively. The pellet was dried and dissolved in resuspension buffer [7 M urea, 2 M thiourea, 56 mM dithiothreitol (DTT), and 2.5% (v/v) CHAPS]. Protein samples were quantified using Bradford assay [75] with BSA as standard. After 1 h incubation in rehydration buffer, 150 mM iodoacetamide was added, and the protein extracts were incubated for an additional 1.5 h to alkylate sulfhydryl groups [28]. Protein samples were further purified using Ready Prep 2-D Cleanup Kit (Bio-Rad, Hercules, CA, USA) following the manufacturer’s instructions. The purified protein was resuspended in IEF resuspension buffer [9 M urea, 4% CHAPS, 0.5% Triton X 100, 20 mM DTT, 1% Ampholyte (pH 3–10)].

### Analytical 2-DE gel electrophoresis

Aliquots containing 400 μg of protein samples were adjusted to a final volume of 315 μl with IEF resuspension buffer. After centrifugation at 15,000 × g to remove insoluble material, the sample was loaded onto an 18 cm immobilized pH gradient strip (pH 4–7; Bio-Rad, Hercules, CA, USA) for passive rehydration. Programmed IEF was performed with the Protean IEF cell (Bio-Rad, Hercules, CA, USA) following the manufacturer’s instructions. Prior to second dimension migration, the strip was equilibrated twice in Equilibration Buffers I and II (Bio-Rad, Hercules, CA, USA), for 15 min each. The second dimension SDS-PAGE was performed with 12% resolving gels and 3.9% stacking gels in a protean II xi cell. The gels were stained with colloidal Coomassie brilliant blue G-250 (CBB) and analyzed as mentioned in [76].

Gel images were acquired using Versa-Doc image system (Bio-Rad, Hercules, CA, USA) and the differentially expressed proteins were identified using the PD-Quest analysis software version 8.0.1 (Bio-Rad, Hercules, CA, USA). The quantity of each spot in a gel was normalized as a percentage of the total quantity of valid spots in that gel. Student’s *t*-test was applied to compare the spots’ relative volume between two groups.

### MS/MS analysis and protein identification

Significant spots with at least a 2.0-fold difference (P < 0.05) were excised and digested with trypsin (In-gel trypsin digestion kit, Pierce, USA). The trypsin-digested peptides were used for MALDI TOF-TOF MS/MS protein identification (MALDI TOF-TOF mass spectrophotometer, ABI-4800 from Applied Biosystems, USA).

Data were interpreted using the GPS Explorer Software (Applied Biosystems), and an automated database search was carried out using the MASCOT program (Matrix Science Ltd., London, U.K.). MS/MS data were used to perform protein identification by searching in a non-redundant protein sequence database (NCBI nr—20070216; 4626804 sequences, 1596079197 residues) using a MOWSE algorithm as implemented in the MASCOT search engine version 3.5 (Matrix science: http://www.matrixscience.com). The following parameters were used for database searches: taxonomy, viridiplantae (green plants; 186963 sequences); cleavage specificity, trypsin with 1 missed cleavage allowed; mass tolerance of 100 ppm for precursor ions and a tolerance of 0.2 Da for the fragment ions; allowed modifications, carbamidomethyl (fixed), oxidation of Met (variable), cleavage by trypsin, cuts C-term side of KR unless next residue is P. According to MASCOT probability analysis, only significant hits with a MASCOT score ≥ 60 (P < 0.05) were accepted.

### Functional annotation

To evaluate the functional categories and hierarchies of identified proteins, KEGG (Kyoto Encyclopaedia of Genes and Genomes, http://www.genome.jp/kegg/) was used [77]. The theoretical peptide mass and pI of the polypeptides were evaluated at EXPASy (http://www.expasy.org/tools/pi_tool.html) [78,79] for final confirmation according to their positions in the 2-DE gel map.

## Competing interests

The authors declare that they have no competing interests.

## Authors’ contributions

DB, SG and AC maintained plant materials and established various cell cultures, DB carried out Lignan extraction and HPLC analysis throughout the studies and SH carried out extraction, fractionation and purification of cell cultures. RS carried out protein extraction, 2-DE, image acquisition and data analysis. SC conceived, designed and implemented this study. All authors read and approved the final manuscript.

## Author information

Plant Biology Laboratory, Drug Development/Diagnostics & Biotechnology Division, CSIR-Indian Institute of Chemical Biology, 4, Raja S. C. Mullick Road, Kolkata-700032, India.

## Supplementary Material

Additional file 1 **The identity of podophyllotoxin as confirmed by mass spectrometry analysis (LC-MS/MS).** The peaks at m/z 437 is attributed to [M+Na]^+^ ion of podophyllotoxin and indicate its presence in the cell suspension cultures. Click here for file

Additional file 2 **Proteins differentially expressed in cells of *P. hexandrum*cell suspension culture elicited with MeJA, as identified by MALDI TOF-TOF MS/MS.** Click here for file

Additional file 3 Sequences of the identified peptides with the corresponding ion score in brackets that were matched based on the MS/MS patterns. Click here for file
