# Supplementary material for: Proteins differentially expressed in elicited cell suspension culture of Podophyllum hexandrum with enhanced podophyllotoxin content
Source: Proteome Sci. 2012 May 23;10:34. doi: 10.1186/1477-5956-10-34 (PMC3499389; doi:10.1186/1477-5956-10-34)
Supplement: Additional file 1 — The identity of podophyllotoxin as confirmed by mass spectrometry analysis (LC-MS/MS). The peaks at m/z 437 is attributed to [M+Na]+ ion of podophyllotoxin and indicate its presence in the cell suspension cultures. [file 1477-5956-10-34-S1.doc]

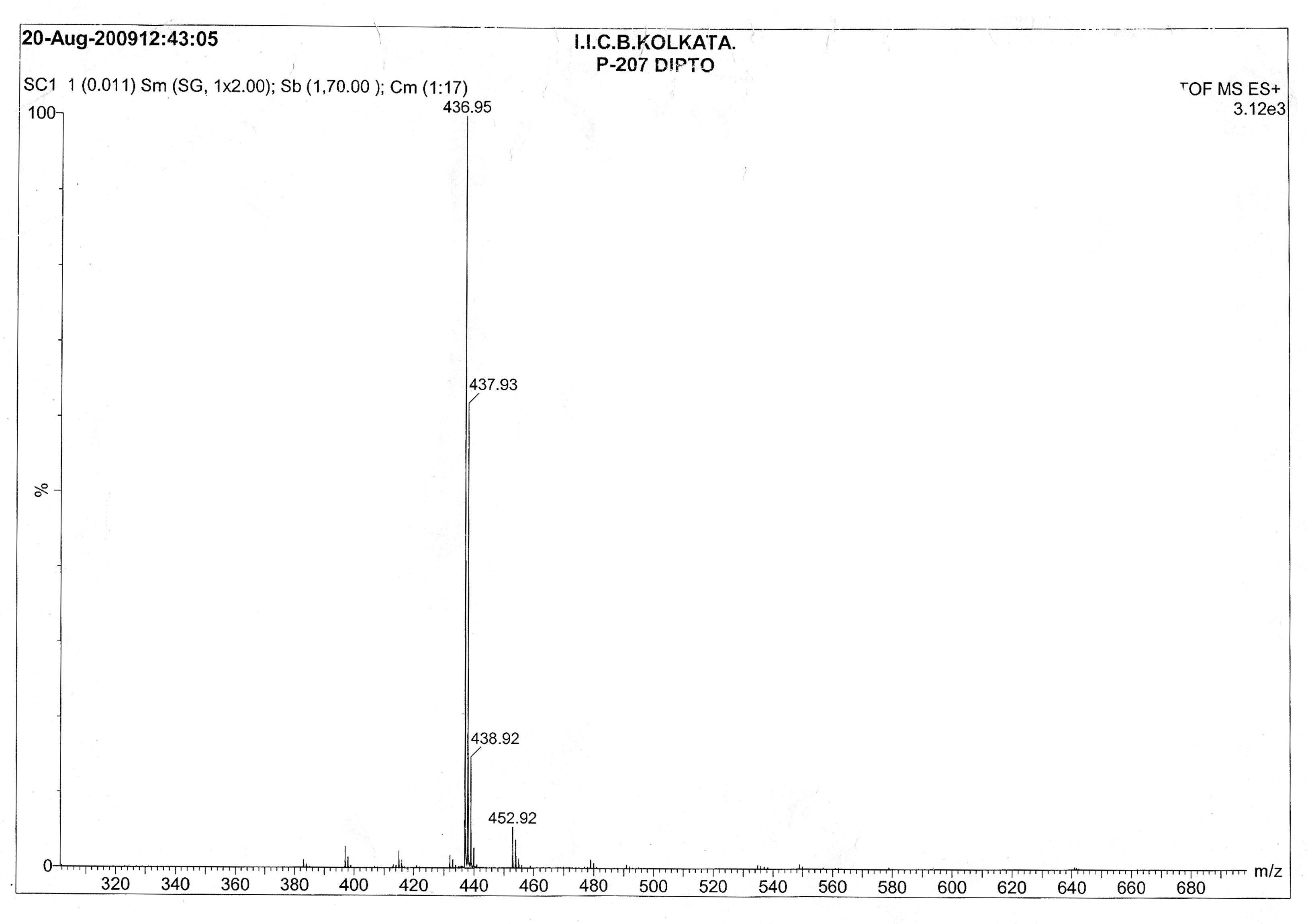


**A**


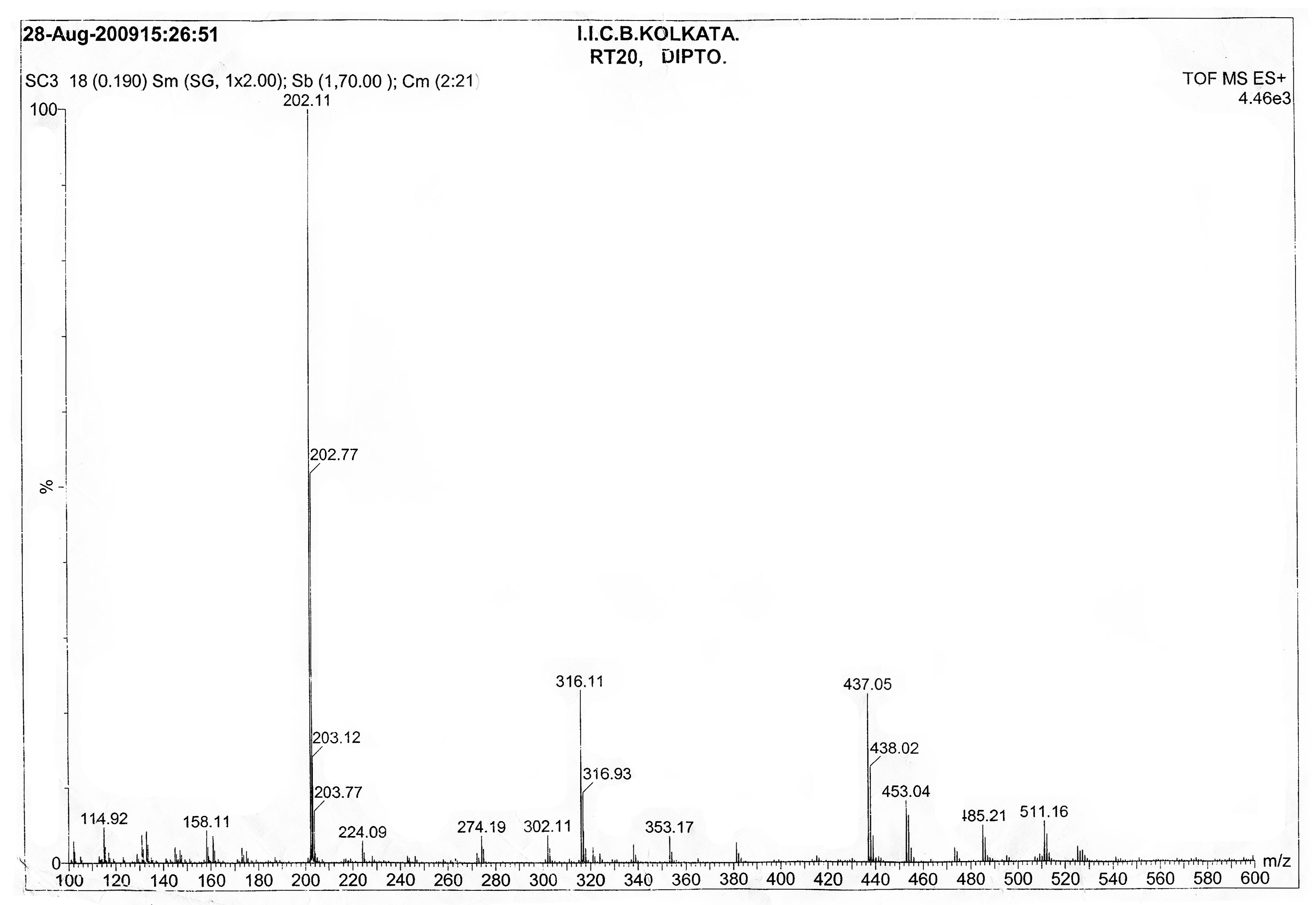


**B**

**Additional file 1.** **The identity of podophyllotoxin as confirmed by mass spectrometry analysis (LC-MS/MS). The peaks at m/z 437 is attributed to [M+Na]^+^ ion of podophyllotoxin and indicate its presence in the cell suspension cultures.**
