# Supplementary material for: Proteins differentially expressed in elicited cell suspension culture of Podophyllum hexandrum with enhanced podophyllotoxin content
Source: Proteome Sci. 2012 May 23;10:34. doi: 10.1186/1477-5956-10-34 (PMC3499389; doi:10.1186/1477-5956-10-34)
Supplement: Additional file 2 — Proteins differentially expressed in cells of P. hexandrumcell suspension culture elicited with MeJA, as identified by MALDI TOF-TOF MS/MS. [file 1477-5956-10-34-S2.doc]

**Additional file 2 – Proteins differentially expressed in cells of *P. hexandrum*** cell suspension culture elicited with MeJA, as identified by MALDI TOF-TOF MSMS.

| **SSP no.*^a^*** | **Fold change*^b^* (mean**±**SD)** | **Protein (Taxonomy)** | **Score*^c^*** | **Accesion no.*^d^*** | **Th. Mr/pI*^e^*** | **Exp. Mr/pI*^f^*** | **S C (%)*^g^*** | **Matched peptides*^h^*** |
| --- | --- | --- | --- | --- | --- | --- | --- | --- |
| **Secondary metabolism** | | | | | | | | |
| 1406 | 4.2±0.0 | Chalcones synthase (*O. viciifolia*) | 62 | gi2565388 | 43.5/6.1 | 44.6/6.3 | 10 | 8 |
| 1403 | ND | Chalcones synthase (*A. halleri*) | 81 | gi13603765 | 1.7/5.8 | 45.5/6.1 | 82 | 3 |
| 1405 | ND | Dioxygenase (*M. macrocarpus*) | 99 | gi1666096 | 36.1/6.2 | 39.6/6.2 | 19 | 6 |
| 8414 | 1.8±0.3 | Tyrosine decarboxylase (*O. poppy*) | 71 | gi1076246 | 40.8/8.6 | 42.1/7.0 | 13 | 7 |
| 1301 | ND | Caffeic acid O-methyl transferase (*P. tremuloides*) | 83 | gi1236980 | 40.1/5.8 | 37.6/5.8 | 14 | 6 |
| 9603 | ND | Polyphenol oxidase (*P. salicina*) | 87 | gi58047496 | 65.8/7.6 | 63.4/4.7 | 6 | 6 |
| 8506 | 1.5±0.6 | Putative serine/threonine protein kinase(*O. sativa*) | 64 | gi55741422 | 45.8/9.6 | 48.1/6.9 | 20 | 8 |
| 9604 | ND | Serine/threonine specific protein kinase NPK5 ( *N. tabacum*) | 74 | gi1076633 | 58.7/8.6 | 58.8/7.2 | 17 | 12 |
| 1301 | ND | Thiazole biosynthetic enzyme (*P. menziesii*) | 63 | gi56481847 | 37.5/5.8 | 37.6/5.8 | 28 | 5 |
| 8412 | ND | Resveratrol synthase *(A. hypogea)* | 61 | gi|7960183 | 43.1/ 5.7 | 44.8/6.9 | 19 | 4 |
| 6202 | ND | Chalcones-flavone isomerase 2 (*M. sativa*) | 72 | gi|116135 | 21.4/ 5.6 | 22.8/6.8 | 36 | 6 |
| 2105 | ND | Caffeoyl CoA 3-O- methyl transferase *(A. sativum)* | 66 | gi|32400289 | 14.7/ 5.5 | 17.5/6.6 | 48 | 2 |
| 8504 | ND | Ent-kaurene oxidase (*O. sativa*) | 69 | gi|48766853 | 57.2/ 5.6 | 57.4/6.9 | 7 | 4 |
| 6418 | ND | Putative orcinol –O- methyl transferase (*R. gigantean*) | 60 | gi|55163140 | 39.1/ 5.6 | 39.5/6.5 | 40 | 3 |
| **Stress and defense related protein** | | | | | | | | |
| 1111 | ND | TSI-1 protein (*L. esculentum*) | 59 | gi2887310 | 20.4/5.6 | 19.4/6.2 | 30 | 4 |
| 8606 | ND | γ-glutamyl transferase (*A. cepa*) | 68 | gi46402910 | 58.4/9.1 | 59.2/6.9 | 14 | 7 |
| 8316 | ND | Putative benzothiadiazole-induced S-adenosyl L-methionine salicylic acid carboxyl methyl transferase (*O. sativa*) | 69 | gi52076850 | 34.6/9.2 | 32.1/6.9 | 23 | 8 |
| 8214 | ND | Pr 1 like protein (*O. sativa*) | 63 | gi50251501 | 27.8/11.6 | 27.2/6.9 | 21 | 5 |
| 8112 | 1.9±0.1 | Antifungal protein 1 (*A. thaliana*) | 65 | gi322464 | 3.1/8.6 | 17.5/6.7 | 92 | 2 |
| 5206 | ND | Glutathione transferaseF5 (*T. aestivum*) | 67 | gi23504745 | 23.4/5.7 | 29.0/6.8 | 31 | 6 |
| 1113 | ND | LRR kinase protein (*G. max*) | 63 | gi47087677 | 5.6/6.8 | 18.1/6.6 | 43 | 2 |
| 2106 | ND | Putative metallothionein (*P. oceanica*) | 70 | gi21668402 | 4.9/4.9 | 17.5/6.6 | 88 | 2 |
| 1102 | 2.3±0.4 | Intracellular pathogenesis related protein PR-104 (*L. longiflorum*) | 78 | gi6649902 | 16.7/5.2 | 19.4/5.4 | 25 | 3 |
| 1114 | ND | NBS/LRR resistance protein like protein (*T. cacao*) | 82 | gi15487902 | 19.9/9.1 | 16.5/6.6 | 23 | 5 |
| 8111 | ND | Leucine rich receptor transmembrane protein kinase 2 (*Z. mays*) | 63 | gi3360295 | 4.5/8.7 | 16.5/7.0 | 88 | 3 |
| 4104 | ND | NBS/LRR resistance protein like protein (*T. cacao*) | 85 | gi15487902 | 19.9/9.1 | 19.8/6.8 | 22 | 5 |
| 9108 | 0.3 ± 0.04 | Pathogenesis related protein -10-3.1 (*P. monticola*) | 63 | gi51317981 | 18.2/5.4 | 17.8/7.0 | 23 | 5 |
| 4407 | 0.2 ± 0.01 | Alcohol dehydrogenase(*T. cacao*) | 80 | gi20506 | 42.2/6.2 | 42.5/6.7 | 22 | 8 |
| 8115 | ND | Pathogenesis related protein -10-3.1 (*P. monticola*) | 67 | gi51317981 | 18.2/5.4 | 18.2/7 | 26 | 6 |
| 1206 | ND | Minor-allergen hazelnut profilin (*C. avellana*) | 70 | gi12659208 | 14.2/4.7 | 30.6/6.2 | 32 | 7 |
| **Signaling** | | | | | | | | |
| 8901 | 2.3±0.3 | Phyocalpain (*Saccharum hybrid cultivar*) | 85 | gi22901868 | 130.5/5.4 | 130.4/6.7 | 6 | 11 |
| 8803 | 2.2±0.5 | Phytocalpain (*Saccharum hybrid cultivar*) | 69 | gi22901868 | 130.5/5.4 | 122.6/6.7 | 12 | 16 |
| 4106 | ND | Thioredoxin like(*O. sativa*) | 80 | gi52076520 | 20.5/9.3 | 20.4/6.8 | 23 | 7 |
| 8605 | ND | Calcium calmodulin dependent protein kinase CaMK 3 (*N. tabacum*) | 94 | gi16904226 | 67.6/8.6 | 67.8/6.9 | 14 | 10 |
| 2609 | 0.4 ± 0.01 | Phosphoinositide-specific phospholipase C family protein (*A. thaliana*) | 99 | gi42565683 | 61.5/8.2 | 61.7/6.6 | 19 | 9 |
| 2608 | 0.2 ± 0.01 | Inositol polyphosphate 5-phosphatase (*A. thaliana*) | 58 | gi42571739 | 68.2/5.6 | 61.7/6.6 | 12 | 10 |
| **Transcription and DNA replication** | | | | | | | | |
| 2203 | ND | S4 RNase (*A. hispanicum*) | 69 | gi1405426 | 27.6/9.2 | 27.5/6.7 | 30 | 5 |
| 4212 | ND | Putative replication protein A1 (*A. thaliana*) | 90 | gi4699943 | 27.9/9.1 | 28.9/6.7 | 31 | 5 |
| 8011 | ND | MLA13UORF 2b (*H. vulgare*) | 61 | gi27464243 | 4.2/11.3 | 15.0/6.9 | 63 | 3 |
| 9003 | ND | Apetala 1 (*E. prenoides*) | 66 | gi45825888 | 4.3/9.5 | 15.0/7.1 | 41 | 2 |
| 4211 | ND | Putative CCR4 associated factor 1 (*O. sativa*) | 72 | gi31429967 | 28.3/5.2 | 28.6/6.7 | 28 | 4 |
| 7316 | 2.2±0.1 | CCAAT-binding transcription factor (CBF-B/NF-YA) family protein (*A. thaliana*) | 80 | gi42572087 | 37.7/9.6 | 37.8/6.9 | 20 | 9 |
| 9104 | 0.7±0.0 | R2R3MYB transcription factor (*A. thaliana*) | 63 | gi2832377 | 5.2/9.7 | 16.5/7.2 | 55 | 4 |
| 7413 | ND | MAR binding protein MFP 1 homologe (*N. tabacum*) | 66 | gi7108717 | 45.7/5.4 | 46.1/6.9 | 21 | 8 |
| 4819 | ND | Pentacotripeptide repeat containing protein (*A. thaliana*) | 71 | gi15241779 | 83.6/7.2 | 90.2/6.8 | 15 | 12 |
| 6510 | ND | MCM2 related protein (*A. thaliana*) | 80 | gi1565223 | 55.1/7.3 | 57.1/6.8 | 23 | 9 |
| 6513 | 0.4± 0.03 | F-box family protein (*A. thaliana*) | 86 | gi15230517 | 51.6/6.0 | 51.3/6.8 | 15 | 9 |
| 6512 | 0.3 ± 0.02 | F-box family protein (*A. thaliana*) | 67 | gi30686368 | 18.8/9.0 | 52.3/6.8 | 32 | 6 |
| 7510 | ND | Maturase (*C. glauca*) | 73 | gi13177529 | 56.8/9.9 | 57.4/6.9 | 20 | 10 |
| 8413 | ND | Pentacotripeptide repeat (PPR) containing protein (*A. thaliana*) | 73 | gi42570837 | 46.1/6.3 | 46.1/6.9 | 17 | 9 |
| **Chloroplast : photosynthesis: electron transfer chain** | | | | | | | | |
| 6105 | ND | Photosystem I reaction centre subunit PSAN precursor (*V. carteri*) | 68 | gi5902586 | 15.9/9.5 | 17.2/6.9 | 41 | 4 |
| 6107 | ND | Photosystem I reaction centre subunit PSAN precursor (*V. carteri*) | 63 | gi5902586 | 15.9/9.5 | 18.1/6.8 | 41 | 4 |
| 7104 | ND | Photosystem I reaction centre subunit PSAN precursor (*V. carteri*) | 72 | gi5902586 | 15.9/9.5 | 16.7/6.9 | 41 | 4 |
| 7319 | 2.6±0.2 | Ferrodoxin-NADP (H) oxidoreductas (*T. aestivum*) | 61 | gi20302471 | 39.2/8.3 | 37.6/6.9 | 19 | 8 |
| **Chloroplast : photosynthesis: carbohydrate pathways** | | | | | | | | |
| 1001 | 0.1±0.0 | Pyrophosphate (*A. thaliana*) | 67 | gi22328511 | 7.3/5.1 | 16.3/6.2 | 53 | 4 |
| 1501 | ND | Glucose 1 phosphate adenyl transferase  (*O. sativa*) | 88 | gi50944557 | 53.2/5.9 | 53.1/6.6 | 16 | 8 |
| **Cytosol : glycolysis and other carbon metabolism enzymes** | | | | | | | | |
| 5315 | ND | Phosphoglycerate dehydrogenase like protein (*O. sativa*) | 97 | gi50510002 | 36.9/5.9 | 36.9/6.8 | 28 | 5 |
| 1104 | 0.6±0.0 | Granule bound starch synthase (*I. hederoccea*) | 68 | gi5052305 | 13.8/5.4 | 16.6/5.7 | 40 | 4 |
| 2101 | ND | Galactinol synthase (*M. charantia*) | 64 | gi34550078 | 2.4/4.5 | 17.2/6.6 | 78 | 2 |
| 4701 | 0.4 ± 0.02 | Galactonolactone dehydrogenase (*B. oleracea*) | 79 | gi7488598 | 68.1/8.7 | 68.6/6.7 | 8 | 8 |
| **Mitochondria: pyruvate pathways and tricarboxylic acid pathway** | | | | | | | | |
| 7302 | 2.1±0.2 | Isocitrate lyase (*P. taeda*) | 69 | gi1353640 | 64.5/7.3 | 36.1/6.9 | 23 | 14 |
| **Mitochondria: electron transfer (oxidative phosphorylation)** | | | | | | | | |
| 0115 | ND | Cyt P 450 (*H. vulgaris*) | 73 | gi57546352 | 3.9/4.9 | 18.9/4.9 | 82 | 2 |
| 7206 | ND | NADH dehydrogenase subunit 9 (*B. vulgare*) | 83 | gi27753503 | 3.9/4.9 | 23.4/6.9 | 82 | 2 |
| 1105 | 1.2±0.1 | Cytochrome C oxidase subunit 5C like protein (*A. thaliana*) | 63 | gi7340711 | 7.0/8.2 | 16.5/5.9 | 35 | 4 |
| 7309 | 0.4 ± 0.02 | Quinone oxidoreductase homologue (*S. oleracea*) | 71 | gi24370984 | 34.8/9.1 | 34.9/6.9 | 20 | 6 |
| 1020 | ND | ATP synthase β (*A. leavisphaera*) | 76 | gi32563485 | 12.9/7.9 | 16.1/6.6 | 45 | 4 |
| 1019 | 5.3±0.3 | Ubiquinol cytochrome C reductase complex 7.8 kda protein (*A. thaliana*) | 63 | gi15218058 | 8.3/7.6 | 16.2/6.6 | 44 | 3 |
| 8805 | ND | Potassium channel protein ZMK2 (*Z. mays*) | 75 | gi5830781 | 94.4/7.6 | 95.0/7.0 | 14 | 13 |
| **Cell wall biosynthesis** | | | | | | | | |
| 1605 | ND | β-glucosidase (*B. napus*) | 98 | gi757740 | 58.9/6.2 | 58.6/5.9 | 9 | 6 |
| **Lipid metabolism** | | | | | | | | |
| 8007 | ND | Putative oleosin protein (*M. scutellata*) | 75 | gi33330959 | 7.9/9.3 | 14.9/6.9 | 30 | 3 |
| 1408 | ND | Myristyl ACP desaturase (*P. hortorum*) | 80 | gi1304409 | 42.3/5.9 | 43.7/6.4 | 24 | 10 |
| **Amino acid and Nitrogen metabolism** | | | | | | | | |
| 6416 | 0.4 ± 0.02 | Putative aspartate aminotransferase (*H. vulgare*) | 76 | gi4102887 | 10.3/5.0 | 45.1/6.9 | 68 | 4 |
| 6417 | ND | Isovaleryl coA-dehydrogenase precursor (*A. thaliana*) | 69 | gi22136242 | 45.3/7.5 | 45.3/6.9 | 21 | 10 |
| 5320 | ND | Putative amino transferase (*O. sativa*) | 68 | gi55168103 | 36.8/6.3 | 36.5/6.8 | 17 | 8 |
| 8401 | 0.8±0.2 | Aspartate aminotransferase (*P. miliaecum*) | 78 | gi20599 | 4.0/7.2 | 44.2/6.9 | 13 | 7 |
| 4408 | ND | Uroporphyrin III C_methyl transferase like protein (*O. sativa*) | 69 | gi34912952 | 40.1/5.8 | 40.1/6.1 | 14 | 6 |
| 3411 | ND | TIN15.8 (At1g48470) (*A. thaliana*) | 80 | gi8778687 | 38.4/6.2 | 38.4/6.7 | 21 | 9 |
| 3409 | ND | Aspartate aminotransferase (*P. miliaecum*) | 73 | gi20599 | 45.0/7.2 | 45.2/6.7 | 17 | 8 |
| 8004 | ND | Glutamine synthase (*S. tuberosum*) | 60 | gi11761905 | 8.9/9.1 | 14.9/6.9 | 62 | 3 |
| 6412 | ND | S adenosyl L-methionine synthetase (*P. hybrida*) | 93 | gi5923879 | 43.0/5.6 | 43.6/6.8 | 22 | 5 |
| **Nucleotide metabolism** | | | | | | | | |
| 8015 | ND | Inosine 5’ monophosphate dehydrogenase (*P. persica*) | 58 | gi321998 | 12.4/9.7 | 15.0/6.9 | 37 | 5 |
| 8005 | ND | Adenosine kinase (*A. tenax*) | 62 | gi19744326 | 10.9/6.8 | 15.0/6.9 | 46 | 3 |
| **Protein translocation, folding, modification, and degradation** | | | | | | | | |
| 6108 | ND | Putative alanine acetyl transferase (*A. thaliana*) | 65 | gi4263715 | 21.2/5.1 | 21.1/6.8 | 30 | 4 |
| 7414 | ND | Translation initiation factor IF-1 (*B. officinalis*) | 69 | gi13774394 | 9.9/9.9 | 47.0/7.0 | 58 | 6 |
| 4508 | ND | Translation elongation factor EF-TuM (*Z. mays*) | 82 | gi11181616 | 48.7/6.0 | 48.2/6.7 | 15 | 9 |
| **Cell cycle** | | | | | | | | |
| 0113 | 0.3 ± 0.01 | Cyclin A like protein (*N. tabacum*) | 66 | gi22324555 | 11.0/4.7 | 17.1/4.6 | 48 | 7 |
| **Hormone biosynthetic enzymes** | | | | | | | | |
| 3604 | ND | 9-cis-epoxy carotenoid dioxygenase-1 (*V. vinifera*) | 65 | gi38112198 | 67.6/6.2 | 67.8/6.7 | 16 | 6 |
| 1408 | ND | Gibberellins 20-oxidase NO3 (*L. sativa*) | 66 | gi9971219 | 43.9/6.1 | 43.7/6.4 | 13 | 8 |
| 4510 | ND | Allene oxide synthase (*S. tuberosum*) | 66 | gi20160364 | 55.6/8.7 | 55.7/6.8 | 16 | 6 |
| 8414 | 2.1±0.6 | Putative 12-oxo phytodieonoic acid reductase (*O. sativa*) | 59 | gi51091133 | 42.6/5.9 | 42.1/6.9 | 21 | 9 |
| **Transposon** | | | | | | | | |
| 7203 | ND | At4g07440 (transposable element gene) (*A. thaliana*) | 64 | gi7267340 | 31.1/5.8 | 30.9/6.9 | 31 | 4 |
| 5513 | ND | Putative transposase (*Z. mays*) | 68 | gi18568261 | 57.3/6.5 | 57.1/6.8 | 12 | 9 |
| **Unknown biological processes** | | | | | | | | |
| 9501 | ND | Hypothetical protein (*O. sativa*) | 76 | gi51536188 | 10.9/8.3 | 54.6/7.2 | 26 | 4 |
| 8706 | ND | Putative protein (*A. thaliana*) | 77 | gi7523418 | 9.7/8.4 | 72.3/6.9 | 48 | 3 |
| 9802 | ND | Hypothetical protein (*O. sativa*) | 68 | gi50938341 | 7.6/11.1 | 90.8/7.6 | 68 | 4 |
| 8004 | ND | Hypothetical protein (*O. sativa*) | 77 | gi55770757 | 6.7/11.6 | 14.9/6.7 | 72 | 4 |
| 7018 | ND | Hypothetical protein (*O. sativa*) | 66 | gi50916395 | 4.8/4.1 | 15.0/6.9 | 74 | 3 |
| 8217 | ND | Unknkown protein (*A. thaliana*) | 69 | gi28827424 | 21.8/5.7 | 23.5/7.0 | 33 | 4 |
| 3112 | ND | Hypothetical protein (*O. sativa*) | 62 | gi57900326 | 7.2/11 | 17.6/6.7 | 56 | 3 |
| 2105 | ND | Hypothetical protein (*O. sativa*) | 79 | gi5.946775 | 16.0/11.8 | 17.5/6.6 | 33 | 5 |
| 1110 | ND | Hypothetical protein (*O. sativa*) | 63 | gi51091495 | 9.0/5.3 | 16.5/5.8 | 69 | 4 |
| 1008 | ND | OSJNBA0013K16.12 (*O. sativa*) | 65 | gi38344280 | 15.9/5.9 | 16.3/5.9 | 29 | 5 |
| 1006 | ND | P0470A12.37 (*O. sativa*) | 66 | gi34906914 | 5.7/10.5 | 16.3/5.7 | 51 | 3 |
| 9109 | ND | P0046E05.2 (*O. sativa*) | 64 | gi34909544 | 16.6/11.4 | 16.7/7.0 | 41 | 5 |
| 6003 | ND | Hypothetical protein (*O. sativa*) | 63 | gi50943681 | 11.6/4.9 | 15.5/6.8 | 46 | 6 |

***^a^***Spot numbers as assigned during PD quest analysis.

***^b^***Fold change in spot accumulation in elicited culture as compared to control. ND: Newly detected, data represented as mean±SD in the three replicate gels.

***^c^***MASCOT score value of the identified protein (p<0.05).

***^d^***NCBI accession number of the identified proteins.

***^e^***Theoretical Mr and pI values of identified proteins as retrieved from the protein database.

***^f^***Experimental Mr and pI values of identified proteins Experimental values were calculated with PD-Quest software and standard molecular mass markers.

***^g^***Amino acid sequence coverage of the identified proteins.

***^h^***Number of matched peptides with respect to total with the PMF data.
