# Supplementary material for: Proteins differentially expressed in elicited cell suspension culture of Podophyllum hexandrum with enhanced podophyllotoxin content
Source: Proteome Sci. 2012 May 23;10:34. doi: 10.1186/1477-5956-10-34 (PMC3499389; doi:10.1186/1477-5956-10-34)
Supplement: Additional file 3 — Sequences of the identified peptides with the corresponding ion score in brackets that were matched based on the MS/MS patterns. [file 1477-5956-10-34-S3.doc]

**Additional file 3. Sequences of the identified peptides with the corresponding ion score in brackets that were matched based on the MS/MS patterns.**

| SSP No. | Matched peptides |
| --- | --- |
| 1406 | R.MDDKSMIK.R 2 Oxidation (M)  R.LGKEAAVK.A K.EAAVKAIK.E  K.EAAVKAIK.E  K.DLAENNK.G  K.DVPGIVSK.N  K.KSAQNGLK.T  K.KSAQNGLK.T |
| 1403 | MVMAAGASSSDEIR.K 2 Oxidation (M)  -.MVMAAGASSSDEIR.K 2 Oxidation (M)  M.VMAAGASSSDEIR.K Oxidation (M) |
| 1405 | K.QRAGPPSPFGYGNK.Q  R.SALNDYIWAVR.N  R.NMACEIVELMAEGLK.I  R.NMACEIVELMAEGLKIQQR.N  R.VLTNSLKSR.I  K.EFTWFEYKR.S |
| 8414 | K.AAQIAGINPK.N  R.SMKLWMVLR.S Oxidation (M)  K.LWMVLR.S  R.FEITVPR.T  R.TFAMVCFRLLPPTTVK.V  R.LLPPTTVK.V  K.LNQVYLR.Q |
| 1301` | K.NPNAPVMLDRILR.L  R.NEDGVSVSPLCLMNQDK.V  K.GVMHVDVIMLAHNPGGK.E Oxidation (M)  K.GVMHVDVIMLAHNPGGK.E Oxidation (M)  R.EFESLAR.G  R.EFESLAR.G |
| 9603` | K.AIELMKALPDDDPR.S Oxidation (M)  K.AIELMKALPDDDPR.S Oxidation (M)  R.YCLYFYEK.I  R.YCLYFYEK.I  K.ISTVVPRPK.Q  R.MWSIWK.T |
| 8506 | R.EWVAEVDFLGQLHHKHLVK.L  R.LLVYEFMARGSLENHLFR.R Oxidation (M)  R.LLVYEFMARGSLENHLFR.R Oxidation (M)  K.GLAFLHGGPKPVIYRDFK.T  R.RALPLPWPCR.M  K.TSNILLDAEYNAKLSDFGLAK.A  K.LSDFGLAK.A  K.LSDFGLAK.A |
| 9604 | R.LFMHPHIIR.L Oxidation (M)  K.GRLQEDEAR.K  R.KFFQQIISGVEYCHR.N  K.FFQQIISGVEYCHR.N  K.FFQQIISGVEYCHR.N  K.GGMISLPSHLSAGAR.D Oxidation (M)  K.TSCGSPNYAAPEVISGK.L  K.GGMISLPSHLSAGAR.D Oxidation (M)  K.GGMISLPSHLSAGARDLIPR.M Oxidation (M)  K.GGMISLPSHLSAGARDLIPR.M Oxidation (M)  R.MLIVDPMKR.M  K.ALQGLNVR.W |
| 1301 | K.VAIIEQSVSPGGGAWLGGQLFSAMVVR.K  K.HAALFTSTIMSK.V  R.MGPTFGAMMVSGQKAAHLALK.A 3 Oxidation (M)  K.VVVSSCGHDGPFGATGVK.R  K.VLARPNVKLFNAVAAEDLIIK.E |
| 8412 | R.VTNSEHMTDLK.K Oxidation (M)  R.EDMMIREVPR.V 2 Oxidation (M)  K.RYMMYHQGCFAGGTVLR.L  R.YMMYHQGCFAGGTVLR.L    R.YMMYHQGCFAGGTVLR.L  R.VLIVCSENTSITFR.G  R.VLIVCSENTSITFR.G  R.DVLSNYGNMSSACVFFIMDLMRK.K 3 Oxidation (M  R.DVLSNYGNMSSACVFFIMDLMRK.K 3 Oxidation (M) |
| 6202 | K.GKSSEELLETLDFYR.D  K.IRELSGPEYSR.K  R.ELSGPEYSRK.V  R.KVMENCVAHLK.S Oxidation (M)  K.VMENCVAHLKSVGTYGDAEAEAMQK.F  K.FAEAFKPVNFPPGASVFYR.Q |
| 2105 | R.VGGLLGYDNTLWNGSVVLPADAPMR.K Oxidation (M)  R.VGGLLGYDNTLWNGSVVLPADAPMRK.Y  K.YIRYYR.D  R.DFVLELNK.A  K.ALAADDRVEICQLPVGDGITLCR.R |
| 8504 | K.TGASPVVVLNSTEVAK.  R.ESMVAISDYGDYQK.M Oxidation (M)  R.LREGDGDK.E |
| 6418 | K.HGYPMTLFELTSALPIHPTK.S  K.LSKTDEEGYTLTDASQLLLK.D  K.GVFEGLESLVDVGGGTGTMAK.A Oxidation (M)  K.YTRGDMFEAVPPADTVLLK.W  K.WILHDWNDQECIK.I  K.WILHDWNDQECIK.I  R.VAITSKDK.K  K.GDEESIETQLFFDMLMMALVGGK.E 2 Oxidation (M)  K.EWAKLFTDAGFSDYK.I  K.LFTDAGFSDYK.I |
| 1111 | R.LFKALVLDFDNLVPK.L  R.LNIDEKESGLHVR.N  K.NNETIEGDGGVGSIKQMNFVEGGPIK.Y Oxidation (M)  R.LNIDEKESGLHVR.N |
| 8606 | K.GMRDIYTTNGTLLMPGDICR.N  R.DIYTTNGTLLMPGDICRNK.K Oxidation (M)  R.EIAVHGPK.V  R.EIAVHGPK.V  R.IPSGVSGSLGLHR.F  R.FIESLKHMMAIR.M  K.LVASLGASGGGMIIAAATHIFLK.L |
| 8316 | K.NIKEPLNEGNIYISK.T  K.DFSNFLELR.S  R.SNELISSGQMLLTFLGRK.N  R.SNELISSGQMLLTFLGRK.N  R.AIQSLVMEGLMEK.K  K.TIIMRSK.L  K.TIIMRSK.L  Oxidation (M)  R.SLRPVLRR.L |
| 8214 | R.STAHPGSTATTGTATGAER.G  R.STAHPGSTATTGTATGAERGGGAAR.V  R.VAETVEGGTGRVYIGLGGGESGR.R  R.VAETVEGGTGRVYIGLGGGESGR.R  K.VRPPLMR.R |
| 8112 | K.LCERPSGTWSGVCGNSNACK.N  K.LCERPSGTWSGVCGNSNACKNQCIN.- |
| 5206 | MAPIKLYGMMLSANVTR.V Oxidation (M)  -.MAPIKLYGMMLSANVTR.V 2 Oxidation (M  -.MAPIKLYGMMLSANVTR.V 2 Oxidation (M)  R.VTTLLNELGLEFDFVDVDLR.T  K.LEAWLEVESHHFYPPAR.T  K.AWWEEISARPAWAK.T |
| 1113 | -.ILSRNQFSGHIPSSIANISSLR.Q  R.NQFSGHIPSSIANISSLR.Q |
| 2106 | .CGCGSGCTCGSGCSGCKMYPDLIEK.C  K.CTSTETLIMGVASEK.R |
| 1102 | R.QLNFSSVMPFSYVK.E  R.LDFIDHEK.F  K.IVPNSSGGCVVKVVTESK.L |
| 1114 | .GMGGVGKTTLLTQINNK.L  K.KATDIFR.K  K.ATDIFRK.L  K.QVAAKCGGLPFAFK.V  K.CGGLPFAFKVLG.- |
| 8111 | K.SGGGGGVGGVAR.R  R.RLLLLACCCIWPGGR.I  R.IFTAADTDPND.- |
| 4104 | -.GMGGVGKTTLLTQINNK.L  K.KATDIFR.K  K.ATDIFRK.L  K.QVAAKCGGLPFAFK.V  K.CGGLPFAFK.V |
| 9108 | -.MVSGTATSEHFSEVEAR.R  -.MVSGTATSEHFSEVEARR.I  K.MVIKYTTIEGGLLGK.K Oxidation (M)  K.YTTIEGGLLGK.K  K.YTTIEGGLLGKK.L  K.EQSNHMFK.K Oxidation (M) |
| 8115 | MVSGTATSEHFSEVEARR.I  -.MVSGTATSEHFSEVEARR.I  K.YTTIEGGLLGK.K  K.YTTIEGGLLGKK.L  K.EQSNHMFK.K Oxidation (M) |
| 1206 | M.SWQAYGDEHLMCEIEGNR.L Oxidation (M)  M.SWQAYGDEHLMCEIEGNR.L Oxidation (M)  K.YMVIQGEPGAVIR.G Oxidation (M)  K.YMVIQGEPGAVIR.G Oxidation (M)  K.YMVIQGEPGAVIRGK.K  K.KGPGGVTVK.K  K.GPGGVTVKK.T |
| 8901 | K.MMEDAVHFLSK.D  R.VSSIANHIR.K  K.MQIMDAFLWGR.C Oxidation (M)  R.DEVNQRMLSVER.A  R.DEVNQRMLSVER.A  R.ISEVIITPEYNDEGIYTVR.F  R.ISEVIITPEYNDEGIYTVR.F  K.LMHVPQAKK.G  K.LMHVPQAKK.G  R.YSVHGQWR.G  R.YSVHGQWR.G |
| 8803 | K.MMEDAVHFLSK.D  K.MMEDAVHFLSKDTVVQAMSR.S 3 Oxidation (M)  R.ADVMKLR.D Oxidation (M)  R.ILALEEAIDTEWVYMWDK.F  R.VSSIANHIR.K  R.VSSIANHIRK.A  R.MTTVAKEWK.N Oxidation (M)  R.SDSEGSDSKMQIMDAFLWGR.C  K.MQIMDAFLWGR.C Oxidation (M)  R.DEVNQRMLSVER.A  R.DEVNQRMLSVER.A  R.ISEVIITPEYNDEGIYTVR.F  R.ISEVIITPEYNDEGIYTVR.F  K.LMHVPQAKK.G  K.LMHVPQAKK.G  R.AAYNIYMHESAGGTDYVNSR.E Oxidation (M) |
| 4106 | M.ALAHRLCR.L  R.AMAPVISKLSSR.Y  K.LSSRYPK.I  K.LSSRYPK.I  K.IPIYKVDIDMDGVGSK.L Oxidation (M)  K.IPIYKVDIDMDGVGSK.L Oxidation (M)  K.VDIDMDGVGSKLSDLK.I Oxidation (M) |
| 8605 | R.HGTVKPNESAIPEGNESEVGDGGGAGLDK.S  K.SKMTTAIAIEDVR.R  K.ILRALTGHNNLVK.F  K.ILRALTGHNNLVK.F  R.LNDIVGSAYYVAPEVLHR.S  K.LSFLGFAK.L  K.LSFLGFAK.L  K.LLHGVSSR.S  K.LSFLGFAKLLHGVSSR.S  K.LLHGVSSR.S |
| 2609 | R.NKYHGYDDDMPNLLPTFIK.L  R.NKYHGYDDDMPNLLPTFIK.L  K.LLDTEKDEDGAGLNAAEQIDR.E  R.SYPVIITIK.D  R.GMLQKALTDDVQQPGWYER.D  R.IGRLSKPDLYVR.I  R.ISIAGVPHDEK.I  R.ISIAGVPHDEK.I  R.AVPLYDERGK.A |
| 2608 | -.MAEVRSR.S  K.IKSHSDPPSPSK.F  R.SLSMQINSDSKR.E  R.IVSKQMVGVFLTIWVR.R  K.QMVGVFLTIWVR.R  K.QMVGVFLTIWVR.R  R.RTQFLPHSLNANELPR.S  K.GMKLFNYR.R Oxidation (M)  K.LFNYRR.N  K.LFNYRR.N |
| 2203 | K.LVLQWPKSFCLINSR.K  R.KHGSCCFPPHESEIYFLK.A  K.TPILKCAQSYLK.E  K.EVVICVDNNGASVVHCPR.S  R.DPCPFSDVK.F |
| 4212 | R.LYELSPLVTGWAICVMVVRVYK.K  R.FSAFYYDRIK.E  R.NDSTSLYQHYKK.T  R.LQSATTNVVVTLYDYLATDLR.L  K.ESASNDYLMIK.L Oxidation (M) |
| 8011 | K.YLINLRSIGAILTPLVR.R  R.SIGAILTPLVR.R  R.CVLLRR.E |
| 9003 | R.KNQLMHESITELQR.K Oxidation (M)  K.NQLMHESITELQRK.E Oxidation (M) |
| 4211 | R.DPHVVDSVEFLKNQGIDFDLAR.Q  R.QIGVTSTAFGEKLLAILPPPSR.R  K.MLTGGQPLPETR.Q Oxidation (M)  K.MLAGHKSVVAAAIFATIR.S |
| 7316 | -.MMHQMLNK.K 2 Oxidation (M)  M.MHQMLNKK.D Oxidation (M)  R.GSAESLSLKVDSRPGHIQTTK.Q  R.GSAESLSLKVDSRPGHIQTTK.Q  R.QISFSAKSGSEITQR.K  R.KGFASNPK.Q  K.RPRGSGGR.F  K.RPRGSGGR.F  R.FEAHMLQNNK.D Oxidation (M) |
| 7413 | K.VLIANLTQAKENLR.R  K.LEVQVTQETLEK.S  K.LQMELEETRTLLQK.N  R.TLLQKNIDETK.R  K.QEKNIVVTLEK.E  K.QISQEARENLEDAHSLVMK.L  K.QISQEARENLEDAHSLVMK.L  K.EEKVEAGEK.A |
| 4819 | K.SESQEFVVVK.F  R.LDSNMKNSQVR.N Oxidation (M)  K.ERLLTEEK.I  R.ANTAWDILSDLMK.N Oxidation (M)  R.MNDLVLKMDEVK.I  K.ADSIHFNTLIDGLCKVGR.L  K.EVVSRMK.E  K.EVVSRMK.E  R.RDHDAIR.V  R.RDHDAIR.V  K.LGNFGQALSLK.E  K.LMDEMVEHLVNQIRSQWR.F |
| 6510 | R.IVERIIK.S  K.TALALAMFGGQEKNIK.G Oxidation (M)  R.GICLIDEFDKMNDQDR.V  K.SFAQNVELTDPILSR.F  K.YLTYSKLYVFPK.L  R.RESMNGQGVSIATR.H Oxidation (M)  R.HLESMIRMSEAHAR.M Oxidation (M)  R.QYVTEEDVNMAIR.V |
| 6513 | R.IVERIIK.S  K.TALALAMFGGQEKNIK.G Oxidation (M)  R.GICLIDEFDKMNDQDR.V  K.SFAQNVELTDPILSR.F  K.YLTYSKLYVFPK.L  R.RESMNGQGVSIATR.H Oxidation (M)  R.HLESMIRMSEAHAR.M Oxidation (M)  R.QYVTEEDVNMAIR.V  K.QAASTSVLSKR.W  R.FPSNVFR.S  K.ILVLDSIWFDRIK.F  K.ILVLDSIWFDRIK.F  K.GGLPMFKNLLVLVFLGNTER.V Oxidation (M)  K.NLLVLVFLGNTERVWK.V  K.NLLVLVFLGNTER.V  K.VFLPLLLEHSPNLTK.L  K.VFLPLLLEHSPNLTK.L |
| 6512 | K.IGVKSAEDYHNCILSCK.E  K.IGVKSAEDYHNCILSCK.E  R.KHLLIMK.K  K.HLLIMKK.C  K.KCLANNNPDAHYIK.G  K.EAIYMYAMLLLCRGR.T 2 Oxidation (M) |
| 7510 | K.SSFLIVKR.L  K.DPSSLHLLR.F  K.INQRFFFFLYNFHVYEYESIFVFLR.N  K.IKLEVFTK.D  K.SILASKGSSLLMNK.W  K.SILASKGSSLLMNK.W  K.GSSLLMNKWK.Y Oxidation (M)  R.FKTSMVR.S  K.TSMVRSQIIENSFLIENASK.K Oxidation (M)  R.LSCAKTLAR.K |
| 8413 | K.AEQVFQEMKELGFLK.G  K.AEQVFQEMKELGFLK.G  R.LHAYSVVSDVEGMEKFLMR.C  R.LHAYSVVSDVEGMEKFLMR.C  R.LALGYKMAGK.M  R.LALGYKMAGK.M  K.MEKAVEK.W Oxidation (M)  K.MEKAVEK.W Oxidation (M)  R.GHISYDQLLYDMNGAGLSWK.I Oxidation (M) |
| 6105 | K.AEQVFQEMKELGFLK.G  K.AEQVFQEMKELGFLK.G  R.LHAYSVVSDVEGMEKFLMR.C  R.LHAYSVVSDVEGMEKFLMR.C  R.LALGYKMAGK.M  R.LALGYKMAGK.M  K.MEKAVEK.W Oxidation (M)  K.MEKAVEK.W Oxidation (M)  R.GHISYDQLLYDMNGAGLSWK.I Oxidation (M)  K.VQAARPAR.A  R.ELLGLGVLLGAAALAPAANAGVVEDLLAK.S  R.LATSYANLARSR.T  K.DAKSCGSK.F |
| 6107 | K.VQAARPAR.A  R.ELLGLGVLLGAAALAPAANAGVVEDLLAK.S  R.LATSYANLARSR.T  K.DAKSCGSK.F |
| 7104 | R.ELLGLGVLLGAAALAPAANAGVVEDLLAK.S  K.VQAARPAR.A  R.LATSYANLARSR.T  K.DAKSCGSK.F |
| 7319 | K.KQEEGVVTNK.F  K.ITADDAPGETWHMVFSTDGEVPYK.K  K.RLVYTNDAGEVVK.G  R.LVYTNDAGEVVK.G  K.MHIQTR.M Oxidation (M)  K.GMEKGIDEIMIPLASK.E  K.GMEKGIDEIMIPLASK.E  K.GIDEIMIPLASK.E Oxidation (M) |
| 1001 | -.MLEITSTMSSVNK.Q Oxidation (M)  -.MLEITSTMSSVNK.Q Oxidation (M  M.LEITSTMSSVNK.Q  K.LISELNEVLAEENTEEKGQWK.K |
| 1501 | R.AYGNNIGGYK.N  R.AYGNNIGGYK.N  R.ATAFGLMK.I Oxidation (M)  R.ATAFGLMK.I Oxidation (M)  K.GEQLKAMMVDTTILGLDDVR.A Oxidation (M)  K.GEQLKAMMVDTTILGLDDVR.A Oxidation (M)  R.ETDGYFIKSGIVTVIK.D  K.EMPYIASMGIYVISKNVMLQLLR.E 3 Oxidation (M) |
| 5315 | R.VLFCGPYWPASTSFTK.E  R.LDSDTIAKASQMK.I Oxidation (M)  K.IIMQYGVGLEGVDVNAATEHK.I  R.IPGSTTGNAVSCAEMAIYLTLGVLR.K  K.VVGGVALKLHSGEPITEVEFVN.- |
| 1104 | K.GSDILYAAISK.F  K.KFEQEIEQLEVMYPDK.A  R.VAKFNVPLAHMNTAGADFMMIPS.- Oxidation (M)  K.FNVPLAHMNTAGADFMMIPS.- 2 Oxidation (M) |
| 2101 | -.MSPAAATETIDSTDAPKR.A  M.SPAAATETIDSTDAPKR.A |
| 4701 | MLRSLLLR.R Oxidation (M)  R.SNARSLRPPFPPLR.T  R.SNARSLRPPFPPLR.T  R.SLRPPFPPLR.T  K.YRVQDSSK.K  K.EAIPAPSPIEQR.W  K.IEIPKDK.E  K.IEIPKDK.E |
| 7302 | -.MAVYVQGASMMMDEEAK.I  -.MAVYVQGASMMMDEEAK.I  K.TASRTFGALDPVQVAMMAK.Y  K.TASRTFGALDPVQVAMMAK.Y  K.TGTELQDVEDK.W  K.WMGMANLRLFSDVVGDAIR.S  K.WMGMANLRLFSDVVGDAIR.S  K.KGMLAYVQQIQR.Q  K.WSGANYYDTLLKTVQGGISGTAAMGK.G Oxidation (M)  K.TVQGGISGTAAMGK.G  K.TVQGGISGTAAMGKGVTEDQFK.A Oxidation (M)  K.TVQGGISGTAAMGKGVTEDQFK.A Oxidation (M)  K.ADAWSSDGRALDLTNHGVVAR.A  R.ALDLTNHGVVAR.A |
| 0115 | R.FMECGSAAAMDYKGNDFSYLPFGPGHSL.- Oxidation (M)  K.GNDFSYLPFGPGHSL.- |
| 7206 | -.MDNQFIFKYSWETLPK.K  -.MDNQFIFKYSWETLPK.K  K.SEHGNRFDTNTDYLFQLLCFLK.L  R.FEVVYNLLSTRYNSR.I  R.ISLVVSLFPSAGWWER.E  R.FEVVYNLLSTRYNSR.I  R.YFDFASPWEQR.N |
| 1105 | K.VAHATLKGPSVVK.E  K.TRTFYDLLER.G  R.TFYDLLER.G  R.TFYDLLER.G |
| 7309 | M.AAKLMHAIQYSGYGGGTDALK.H Oxidation (M)  -.MAAKLMHAIQYSGYGGGTDALK.H  R.KFPTIPGTDVAGEVVQAGSAVNR.F  K.FPTIPGTDVAGEVVQAGSAVNR.F  K.GLGADEVLDYK.T  K.QLVPLLLIPK.I |
| 1020 | R.AFGTSTLIAKNIGR.I  K.NIGRITQIIGPVLDVTFSPGK.M  K.MPNIYNSLIVK.G Oxidation (M)  K.VRAVAMSATDGLMR.G Oxidation (M) |
| 1019 | M.ADDEVVDPK.K  K.CVKPLLEYQACVK.R  R.IQGDDSGHK.H |
| 8805 | R.ENAVYSMLGVLRLWR.L  R.AESLNQQQLMDQLPKSICK.S  R.GVSREMLLSLVTK.M Oxidation (M)  R.GVSREMLLSLVTK.M Oxidation (M)  R.EMLLSLVTK.M  K.EAMQSRPEDSVVVIK.N Oxidation (M)  K.EAMQSRPEDSVVVIK.N Oxidation (M)  R.RGHLGALQELLK.L  R.RGHLGALQELLK.L  R.LQSTSSDSQRWPR.V  K.GHPFLRNR.T  R.DNDKLFMVTEEDLR.R Oxidation (M)  R.RLASMDWLSCE.- Oxidation (M) |
| 1605 | R.MSIAWPR.I Oxidation (M)  K.GVSQAGVQFYHDLIDELK.R  K.GVSQAGVQFYHDLIDELKR.N  R.SLLKYIK.D  K.YYLQRHLLSMNEAICIDK.V Oxidation (M)  K.NRFGLYYIDFK.N  R.FGLYYIDFK.N  R.FGLYYIDFK.N  R.FGLYYIDFKNNLTR.Y  R.YEKESGR.Y  K.DFLSQGVRPSMINR.D  K.DFLSQGVRPSMINR.D |
| 8007 | R.DLQAAVPGRMADVFDYVGQK.T Oxidation (M)  R.MADVFDYVGQK.T  R.MADVFDYVGQKTK.D |
| 1408 | R.TGVYTVMDYINILEHFVEK.W Oxidation (M)  R.TGVYTVMDYINILEHFVEK.W Oxidation (M)  R.KVGVTGVMAPQK.I Oxidation (M)  R.KVGVTGVMAPQK.I Oxidation (M)  K.SMEEWGKHNILPLAKPVEK.S Oxidation (M)  K.LDMRQVEK.T  K.LDMRQVEK.T  K.QLAQICGTIAADEKR.H  K.QLAQICGTIAADEKR.H  K.LFELDPDETMSCLAHMMK.R 3 Oxidation (M) |
| 6416 | -.DGRPVVLDCVR.E  R.EAECRIAGNLNMEYLPMGGSIHMIEESLK.L 3 Oxidation (M)  K.RIAAVQALSGTGACR.L  R.LFADFQK.R |
| 6417 | R.SILGYAVK.T  R.SILGYAVK.T  K.AGSKGITAFIIEK.G  K.AGSKGITAFIIEK.G  K.GMTGFSTAQKLDK.L  K.LDKLGMR.G Oxidation (M)  K.EGKGVYVLMSGLDLER.L  K.VADMYTALQSSRSYVYSVAR.D Oxidation (M)  R.DAKLYEIGAGTSEIR.R  K.LYEIGAGTSEIR.R |
| 5320 | K.LQEKWNSTK.H  R.AILVQMTAASKCR.N  R.AILVQMTAASKCR.N  R.LVDAGVLR.S  R.LVDAGVLR.S  R.LVDAGVLRSVGAAR.I  K.IALALSDMLCEDIK.A Oxidation (M)  R.VLVPYDQAS.- |
| 8401 | R.VATVQCLSGTGSLR.V  R.VATVQCLSGTGSLR.V  K.DSEMFHEWTVELKAMADR.I Oxidation (M)  K.DSEMFHEWTVELKAMADR.I Oxidation (M)  K.AMADRIISMR.Q Oxidation (M)  R.IISMRQQLFDALK.S  R.ISMAGLNMK.N 2 Oxidation (M) |
| 4408 | R.DDEIALQLPELRR.L  R.DDEIALQLPELRR.L  R.FLTGHSR.N  R.GTTPQQRMVFAMLK.D 2 Oxidation (M)  K.DFVNEVK.S  K.VVALSPFWIDSSK.Q |
| 3411 | -.MTSPLSDLLNLDLSDTKK.I Oxidation (M)    M.TSPLSDLLNLDLSDTK.K  K.KIIAEYIWIGGSGMDIR.S Oxidation (M)  K.KIIAEYIWIGGSGMDIR.S Oxidation (M)  R.TLPGPVSNPTKLPK.W  R.TLPGPVSNPTKLPK.W  K.QHIAAYGEGNERR.L  R.GASVRVGR.D  R.DTEKEGK.G |
| 3409 | R.AEQMLINDPSRVK.E  R.VATVQCLSGTGSLR.V  R.VATVQCLSGTGSLR.V  K.DSEMFHEWTVELKAMADR.I Oxidation (M)  K.DSEMFHEWTVELKAMADR.I Oxidation (M)  K.AMADRIISMR.Q Oxidation (M  R.QEYHIYMTSDGR.I Oxidation (M)  R.ISMAGLNMK.N 2 Oxidation (M) |
| 8004 | K.EHIAAYGEGNERR.L  R.GASVRVGR.D  K.AGKGYFEDRPASNMDPYVVTSMIAETTIVG.- Oxidation (M) |
| 6412 | R.DTCRGIGFTSADVGLDADHCK.V  R.VHTILISTQHDETVTNDQIAQDLK.E  R.SGAYIVRQAAK.S  K.DVLTLIK.E  K.ENFDFRPGMMSINLDLLRGGNFR.Y 2 Oxidation (M) |
| 8015 | R.GMGSLEAMTK.G 2 Oxidation (M)  R.GMGSLEAMTK.G 2 Oxidation (M)  K.IAQGVVGAVADK.G  R.LEVRTGAAQAEGGVHGLVSYEK.K  R.TGAAQAEGGVHGLVSYEK.K |
| 8005 | K.FLYSAGFFLTVSPESMLTVAK.H  K.YYMINLAAPFICQFFK.D  K.MAALPKAI.- |
| 6108 | -.MEMDSTTTIKPETLSTVSSSPPEK.I  K.GIATEAVR.L  R.LVAGEIFKEKPEMQR.L  K.VGFVKEGVMR.K Oxidation (M) |
| 7414 | K.FGMKEEK.W  K.WIHEGLITESLPNGMFR.V  R.VRLDNEDLVLGYVSGK.I  R.LDNEDLVLGYVSGKIR.R  R.YDSTRGR.I  R.YDSTRGR.I |
| 4508 | R.TKPHVTVGTIGHVDHGK.T  R.TKPHVTVGTIGHVDHGK.T  R.HYAHVDCPGHADYVK.N  0 K.EHILLAR.Q  K.EHILLAR.Q  K.FPGDEIPIIR.G  R.GQVVCRPGYSEDCK.K  R.GQVVCRPGYSEDCK.K  R.TVGAGVVSK.V |
| 0113 | R.GVLVDWLVEVAEEYK.L  R.QKLQLLGVSSMLIASK.Y Oxidation (M)  K.LQLLGVSSMLIASK.Y Oxidation (M)  K.LQLLGVSSMLIASK.Y Oxidation (M)  K.EDVVKMEADVLQSLK.F  K.EDVVKMEADVLQSLK.F  K.MEADVLQSLK.F |
| 3604 | K.TTLPKEK.D  R.VQIAGNFAPVPEQPVQHSLPVDGR.I  K.SPDVEIPLAEPTMMHDFAITER.F Oxidation (M)  K.SVLSEIR.L  K.STRRPILPESEQVNLEAGMVNR.N Oxidation (M)  R.YGGEPLFLPRDPNSGR.E |
| 1408 | -.MPPFLLPSPSR.L  -.MPPFLLPSPSR.L  M.PPFLLPSPSRLPK.K  M.PPFLLPSPSRLPK.K  R.KSLAFFLSPR.M  R.KSLAFFLSPR.M  R.YPDFTWSTFLEFTQK.H  K.NTLDAFSNWLK.N |
| 4510 | M.ALTLSFSLPLPSLHQQFPSK.Y  K.YKSTIFR.T  K.DLFTGTYMPSTELTGGYR.V  R.RDHVIPK.F  K.GTAGLNSGNDQAAFNFLAR.S  K.AGVEVHTR.L |
| 8414 | K.DAAAAAEIPLLTPYKMGR.F Oxidation (M)  R.VVLAPLTR.N  R.SYGNVPRPHAVLYYTQR.A  R.QITPDDSGIVYSKPR.R  R.QITPDDSGIVYSKPRR.L  R.TEEIPQIIDDFR.R  R.TEEIPQIIDDFR.R  R.HFLANPDLPK.R  R.HFLANPDLPKR.F |
| 7203 | K.NNRVDATIPQGNYHNPFRPFLKPGTWIHISGFR.V  R.IYLNPTNFPEIDIQSYIR.G  R.GFEDYDELPENPRMK.V Oxidation (M)  K.CISSAAYNYQPIVAIVR.F |
| 5513 | K.NLIETFRTAISFLNSSNQR.I  K.NLIETFRTAISFLNSSNQR.I  K.SYCIATK.I  K.SYCIATK.I  R.KFQLDMEVR.W  R.KFQLDMEVR.W  K.YWRNIPLLYSFAFILDPR.A  R.AKLTDVYNK.Y  R.AKLTDVYNK.Y |
| 9501 | K.TMVDCLATFVEEMQFQIK.L 2 Oxidation (M)  K.TMVDCLATFVEEMQFQIK.L 2 Oxidation (M)  K.TMVDCLATFVEEMQFQIKLILK.I |
| 8706 | M.GKTISFSAIILVFLLVSTGLMK.Q Oxidation (M)  K.YEGAISGVCVSDPHR.C  K.YEGAISGVCVSDPHRCLCR.N |
| 9802 | -.MAQLSRVWVAATVAAVR.A Oxidation (M)  R.VAGLAPQAAALAAARAAADADGR.R  K.AMYLSCWGPS.-  K.AMYLSCWGPS.- |
| 8004 | M.VAGSKELAAAMAPVTRPR.W Oxidation (M)  M.VAGSKELAAAMAPVTRPR.W Oxidation (M)  R.WSVLPAPPR.R  R.WPPPPPAGIAAVCFGYR.R |
| 7018 | M.PPNLTGMEVAER.E  R.DGEEMKEGK.E Oxidation (M)  K.EEELVADMPFF.- |
| 8217 | K.KYGCDAEVSIHAYANDNTVSVTMR.R Oxidation (M)  K.YGCDAEVSIHAYANDNTVSVTMRR.Q  R.QFSAAGIKLEVFTQAGDK.Y  R.LFPVGSYPSFLAAIFDGGLPK.S |
| 3112 | -.MIEHNFTNHK.S Oxidation (M)  R.GPSGWGSAAAGEVWR.M  R.GPSGWGSAAAGEVWRMR.C  R.GPSGWGSAAAGEVWRMR.C  R.CVGEGRVATGCADVPSAAAGR.R |
| 1110 | -.MDEVGGRWTFSR.A Oxidation (M)  M.DEVGGRWTFSR.A  R.ANEGYTSCGPVVEMSWQRDGLGTMSR.L  R.SFGEDVLGGVLAYTPVER.L |
| 1008 | -.MESGSESGSGSGSR.G  R.EQIEMDDEMER.K 2 Oxidation (M)  R.EQIEMDDEMER.K 2 Oxidation (M)  R.EVESTPRPPK.A  K.ARAGSILAG.- |
| 1006 | M.AVEAVVASLTAAVIRR.F  R.RLSVDDVDPR.R  R.LSVDDVDPRR.G |
| 9109 | K.TRTSDLR.N  R.AAAAWPGAK.T  K.TWFSPEEKR.F  K.RFLSHSAATTIQKPSTR.G  K.NGSPLPHTSLLDVAIFYRQR.R |
| 6003 | R.EVMIFPEIR.L  R.LFNGILGYVR.R  R.LFNGILGYVRR.C  R.EAIAGGGTRFQWR.F  R.EAIAGGGTRFQWR.F  R.TKQGASEIDDMSVQDLV.- Oxidation (M) |
